# Early Activation of Pulmonary TGF-**β**1/Smad2 Signaling in Mice with Acute Pancreatitis-Associated Acute Lung Injury

**DOI:** 10.1155/2014/148029

**Published:** 2014-02-12

**Authors:** Hamid Akbarshahi, Asha Sam, Chaolei Chen, Ann H. Rosendahl, Roland Andersson

**Affiliations:** ^1^Department of Surgery, Clinical Sciences-Lund, Lund University and Skåne University Hospital, 221 85 Lund, Sweden; ^2^The First Affiliated Hospital of Wenzhou Medical University, Wenzhou 325000, China; ^3^Department of Oncology, Clinical Sciences-Lund, Skåne University Hospital and Lund University, 221 85 Lund, Sweden

## Abstract

Acute lung injury is caused by many factors including acute pancreatitis. There is no specific therapy directed at underlying pathophysiological mechanisms for acute lung injury. Transforming growth factor-**β** (TGF-**β**) is involved in the resolution of lung injury in later phases of the disease. Some evidence exists demonstrating that TGF-**β** not only is involved in the late stages, but also contributes to lung injury early on in the progress of the disease. Acute pancreatitis was induced using ductal ligation in mice. TGF-**β**1, 2, and 3, T**β**RII, ALK-5, Smad2, 3, 4, and 7, and P-Smad2 expression in the lungs were analyzed at 9 and 24 h. We demonstrate that TGF-**β**1 levels in the lungs of mice with acute pancreatitis increase as early as 9 h after induction. We observed an increased expression of ALK-5 in acute pancreatitis at both 9 and 24 h. Inhibitory Smad7 expression was transiently increased at 9 h in acute pancreatitis, but reduced later at 24 h, with a concomitant increased nuclear translocation of phosphorylated Smad2. Our findings demonstrate activation of TGF-**β** signaling in the lungs as early as 24 h after acute pancreatitis, suggesting that TGF-**β** may represent a potential therapeutic candidate in acute pancreatitis-induced acute lung injury.

## 1. Introduction

Acute lung injury (ALI) is a devastating syndrome and an important cause of mortality in critically ill patients. The syndrome is characterized by hypoxemia and respiratory failure due to exudation to alveolar spaces which impairs gas exchange. The risk factors for developing this syndrome include pneumonia, gastric aspiration, sepsis, shock, and acute pancreatitis. Although acute pancreatitis represents one of the less common clinical disorders associated with acute respiratory distress syndrome (ARDS), severe attacks of pancreatitis are frequently associated with acute lung injury and respiratory failure [1]. The underlying molecular mechanisms behind the development of lung injury are not fully understood, which may explain the lack of specific pharmacologic therapies.

Transforming growth factor *β* (TGF-*β*) is a multifunctional cytokine regulating inflammatory and fibrotic disorders. It plays a critical role in embryonic development as well as in the resolution of tissue injury in multiple organs, including the lung [2]. TGF-*β* exists in three isoforms and is a member of a large family of soluble proteins that modulate several cellular processes [3]. Of these isoforms, TGF-*β*1 is generated in greatest abundance subsequent to tissue damage [4]. TGF-*β* signaling is initiated via ligand-induced heteromeric complex formation of the TGF-*β* type I and type II serine/threonine kinases receptors. Upon ligand binding, the TGF-*β* type II receptor (T*β*RII) phosphorylates the type I receptor, which then propagates the signal through phosphorylation of the Smad protein cascade. Smad proteins constitute three functional classes: the receptor-regulated Smad (R-Smad), the Comediator Smad (Co-Smad), and the inhibitory Smad (I-Smad). R-Smads (Smad1, 2, 3, 5, and 8) are directly phosphorylated and activated by the type I receptor kinases and form heteromeric complexes with the Co-Smad, Smad4. The activated Smad complexes are translocated into the nucleus and, in conjunction with other nuclear cofactors, regulate the transcription of target genes. The I-Smad, Smad6 and Smad7, negatively regulates TGF-*β* signaling by competing with R-Smads for receptor or Co-Smad interaction and by targeting the receptors for degradation [5].

TGF-*β* has been most thoroughly evaluated for its crucial role in the development of pulmonary fibrosis and airway remodeling during the late phases of chronic lung injury [6, 7]. However, the involvement and regulation of TGF-*β* in acute lung injury are largely unknown. Murine models have demonstrated that the expression levels of several TGF-*β*-responsive genes involved in extracellular matrix modulation and fibrinolysis were dramatically increased in the early phase after induction of lung injury [8]. In addition, elevated levels of TGF-*β*1 as well as TGF-*β*-inducible genes, such as procollagen type III and *α*1, have been demonstrated in the lungs of patients with ARDS [9, 10]. Procollagen III is one of the earliest predictors of the severity of ALI [11–14].

TGF-*β* has been shown to directly increase alveolar epithelial permeability by increasing the gaps between the endothelial cells [15–18]. Increased epithelial permeability permits migration of neutrophils, which stimulates repair of the pulmonary epithelium. Epithelial injury and repair are essential in determining the clinical fate. However, the regulating steps for the injury and repair are incompletely understood [19].

We hypothesized that TGF-*β* signaling might be active early in the lungs in ALI and plays a significant part in the flooding of the alveolar spaces and lung injury. The aim of the present study was to investigate the early activation of TGF-*β* signaling in the lungs of a murine model of acute pancreatitis-associated ALI.

## 2. Material and Methods

### 2.1. Antibodies

Antibodies against TGF-*β*1-3, T*β*RII, and ALK5 (T*β*RI) were from Santa Cruz Biotechnology Inc. (Santa Cruz, CA, USA). Antibodies against Smad2, 3, 4, and 7 and P-Smad2 were kindly provided by professors Carl-Henrik Heldin and Peter ten Dijke. The antibodies were generated and purified as previously described [20].

### 2.2. *In Vivo* Model

*Animals.* 8–10 -week- old male wild-type C57BL/6 mice were purchased from Charles River, Germany. The mice were housed in appropriate facilities at Lund University, under specific pathogen-free conditions and handled according to the institute guidelines with approval of the Malmo-Lund Animal Care Ethics Committee. The animals were kept under 12/12 h light/dark regime in standard mesh cages with laboratory chow and drinking water ad libitum.

*Animal model.* Acute pancreatitis was induced using the combined pancreatic duct and bile duct (BPD) ligation model as described previously [21]. The BPD ligation model is a highly acute model that elicits a pronounced pulmonary inflammatory response as early as 9 h after acute pancreatitis induction [21]. Briefly, the mice were anesthetized and maintained with 2–4% isoflurane. Under aseptic conditions, a midline laparotomy was performed. The bile duct, proximal to its entry into the pancreas, and the common bile-pancreatic duct, near its junction with the duodenum, were dissected and ligated (BPD group). The same procedure was applied to sham-operated control mice where the common bile-pancreatic duct and the bile duct were dissected, but not ligated, after which the abdomen was closed. The mice recovered rapidly after surgery, and postoperative buprenorphine analgesia (0.05 mg/kg, s.c.) was administered twice daily. The animals (*n* = 8 in each group) were sacrificed by exsanguination through puncture of the abdominal aorta 9 and 24 h after pancreatitis-induced surgery. Lung biopsies were harvested, fixed in 4% paraformaldehyde for further immunohistochemical processing or snap-frozen in liquid nitrogen, and stored at −80°C until Western blot analyses.

### 2.3. Immunohistochemistry

Paraffin embedded tissues were sectioned 4 *μ*m thick. Endogenous peroxidase activity was quenched for 30 min in darkness by 1% H_2_O_2_ (Sigma Aldrich, St. Louis, MO, USA) in methanol (Histolab, Gothenburg, Sweden). After blocking with avidin-biotin (Vector Laboratories, Burlingame, CA) and normal goat serum (5%) for 60 min, the sections were incubated with primary antibodies in Tris-buffered saline (TBS)/saponin buffer overnight. Biotinylated goat anti-rabbit IgG secondary antibodies were added in 2% normal blocking serum. Slides were developed for 5 min in diaminobenzidine (DAB, VECTASTAIN, Vector Laboratories) and counterstained with Mayer's hematoxylin (Histolab). Images were analyzed using the ScanScope CS GL SS5082 and Aperio ImageScope v11.1.2.760. Positively stained cells were quantified as average number of cells in 10 microscopic fields (20x magnification) by two researchers blinded to the samples.

### 2.4. Western Immunoblotting

Snap-frozen lung tissue was homogenized in liquid nitrogen using a mortar and pestle. Proteins were extracted in 1% TritonX-100, 10 mM Tris-HCl, 50 mM NaCl, 5 mM EDTA, 30 mM sodium pyrophosphate, 50 mM NaF, 0.1 mM Na_3_VO_4_, and complete protease inhibitor cocktail (Sigma). Protein concentration was determined using BCA protein assay reagent kit (PIERCE ThermoScientific). Lysates were dissolved in Laemmli buffer, boiled and separated by SDS-PAGE (12%), and transferred to 0.2 *μ*m Hybond-C extra nitrocellulose membrane (Amersham Biosciences). Blots were blocked in 5% (w/v) milk in Tris-buffered saline Tween-20 (TBST) and incubated overnight at 4°C with the primary antibodies (1 *μ*g/mL). Following washing, the membranes were incubated for 1 h with the secondary HRP-linked goat anti-rabbit antibody at 1 : 2000. Immunoblotted proteins were visualized by SuperSignal West Dura Substrate (PIERCE ThermoScientific) using LI-COR Odyssey Fc Imager (LI-COR Biosciences) and Image studio Vr 2.0 software.

### 2.5. Statistics

The data is presented as mean ± SEM. Statistical analyses were performed by two-tailed Student's *t*-test using Prism software. A  *P*  value of <0.05 was considered statistically significant. Densitometry analysis was performed using Image studio Vr 2.0 software.

## 3. Results

### 3.1. Increased TGF-*β*1 Expression in the Lungs following Acute Pancreatitis

**Figure 1:**
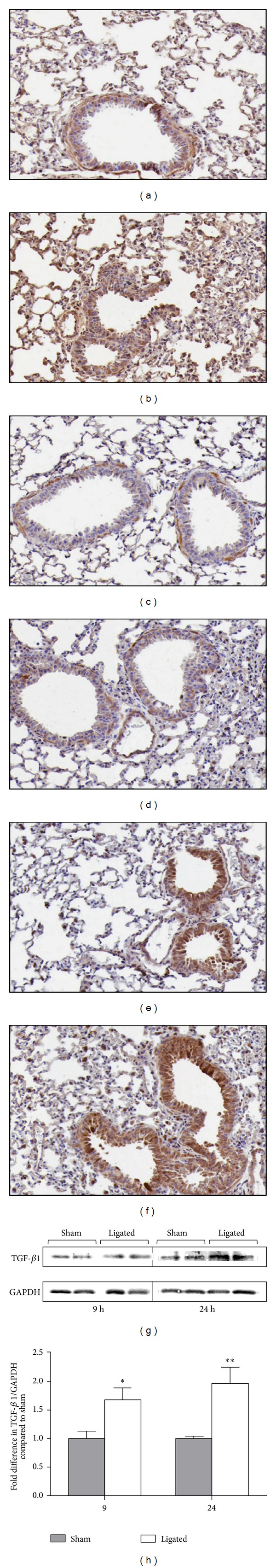
Expression of three different isotypes of TGF-*β* in the lungs. Immunostaining of TGF-*β*1 (a and b), TGF-*β*2 (c and d), and TGF-*β*3 (e and f) levels in lung tissue from sham controls (a, c, and e) and 9 h after acute pancreatitis induction (b, d, and f). Positive staining is visualized in brown by DAB and counterstained in blue by haematoxylin. Results from one representative of eight samples per group are shown. Original magnification, ×20. Western blot analysis of TGF-*β*1 protein levels in lung extracts of sham-operated and ligated animals at time points indicated (g). Two of five similar analyses per group are shown. Graph shows densitometry evaluation of fold difference in TGF-*β*1 intensity compared to sham control and represents mean ± SEM of five samples per group (h). *P < 0.05; **P < 0.01.

To investigate the role of the TGF-*β* system in the progression of ALI due to acute pancreatitis, levels of TGF-*β*1, -*β*2, and -*β*3 were examined in the lungs of ligated animals with acute pancreatitis compared to sham. In the lungs of the animals with AP, TGF-*β*1 was associated predominantly with the bronchial epithelium and macrophages at both time points. Smooth muscle cells and endothelial cells were stained. Occasional fibroblast-like cells and type II alveolar epithelial cells were also stained (Figure 1(b)). There was a 1.68-fold enhanced expression of TGF-*β*1 in the lungs of this group compared to the sham-operated animals (*P* < 0.05; Figures 1(a), 1(b), 1(g), and 1(h)). These changes were more pronounced after 24 h as compared to 9 h (*P* < 0.01; Figures 1(g) and 1(h)).

Staining for TGF-*β*2 in airways of the animals with AP showed similar pattern to that for TGF-*β*1. Bronchial epithelial cells and smooth muscle cells were stained more intensely compared to other cell populations and fibroblast-like cells were stained more consistently compared to TGF-*β*1. Neutrophils were positive for TGF-*β*2 as well as macrophages, sub-epithelial fibroblast-like cells which were stained moderately. No obvious difference between the sham-operated and ligated animals at any of the time points (Figures 1(c) and 1(d)).

TGF-*β*3 staining was also predominantly associated with bronchial epithelial cells. Macrophages, fibroblast-like, and smooth muscle cells were also showing expression. In the ligated group, macrophages were generally stained; subepithelial fibroblast-like cells showed mixed but predominantly positive staining. The bronchial epithelial cells showed a stronger staining tendency in the ligated group in both 9 (Figures 1(e) and 1(f)) and 24 h (data not shown).

Protein extracts from the lungs of the sham-operated and ligated animals were analyzed by Western blotting. Total TGF-*β*1 protein levels were higher in the lung extracts of the ligated animals at 9 and 24 h compared to the lungs of the sham-operated group (Figures 1(g) and 1(h)). There was not any difference for TGF-*β*2 and 3 protein levels between the sham-operated animals and the acute pancreatitis group (data not shown). These results indicate that early modulation of TGF-*β* ligands in the lungs of mice with acute pancreatitis mostly relates to induction of the TGF-*β*1 isoform rather than TGF-*β*2 and TGF-*β*3.

### 3.2. High Expression of ALK5 in the Lungs of the Animals with Acute Pancreatitis

**Figure 2:**
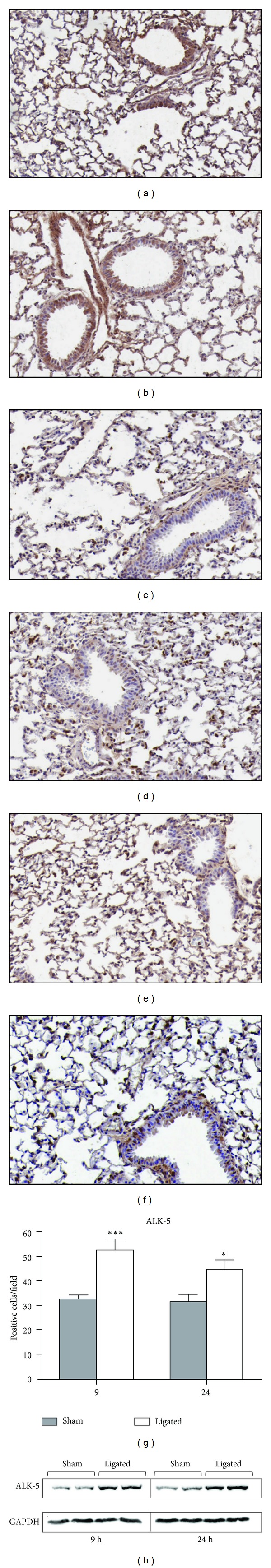
Expression level of the TGF-*β* receptors. Representative images of the levels of T*β*RII at 9 h (a and b) and ALK5/T*β*RI at 9 h (c and d) and 24 h (e-f), and in lung tissue from sham controls (a, c, and e) and after acute pancreatitis induction (b, d, and f). Results from one representative of eight samples per group are shown. Original magnification, ×20. Number of ALK5-positive cells in the lungs of sham controls and ligated groups at 9 and 24 h (g). Graph represents number of positive cells as mean ± SEM in 10 microscope fields (×20 magnification), *n* = 8 per group. *P < 0.05; ***P < 0.001 versus sham control, two-tailed Student's *t*-test. Western blot analysis of total ALK5 protein levels in lung extracts of sham-operated and ligated animals at the early 9 h and advanced 24 h time points (h). Two of five similar analyses per group are shown.

To assess modulation of two candidate receptors for TGF-*β* signaling, the lung sections were stained for T*β*RII and ALK5 (T*β*RI). Both the acute pancreatitis (Figure 2(b)) and sham-operated groups (Figure 2(a)) demonstrated high levels of T*β*RII in bronchial epithelial and smooth muscle cells, while alveoli, fibroblasts, endothelial, and infiltrating cells showed moderate to weak staining (Figures 2(a) and 2(b)). No obvious difference in the expression pattern of T*β*RII was observed between the acute pancreatitis group and sham controls at the time points studied.

Bronchial epithelial and vascular endothelial cells were strongly positive for ALK5, while fibroblasts and alveoli were moderately positive for ALK5 in the lungs of the pancreatitis-induced group (Figures 2(d) and 2(f)). A similar staining pattern was observed in the lungs of the sham-operated groups (Figures 2(c) and 2(e)). Interestingly, the bronchial epithelial cells demonstrated an enhanced nuclear accumulation of ALK5 after 9 h that was further enhanced at 24 h after acute pancreatitis induction compared to sham controls. In addition, acute pancreatitis following BPD-ligation was further associated with enhanced number of infiltrating cells that were more ALK5-positive compared to sham (Figures 2(d) and 2(f)). A significant overall increase in the number of ALK5-positive cells per field was found in the pancreatitis-induced groups compared to sham at both 9 and 24 h (Figure 2(g), 53 versus 32; *P* < 0.001 and 45 versus 32; *P* < 0.05; resp.).

The elevated ALK5 levels in the lungs following acute pancreatitis induction were further confirmed by Western blot of total protein extracts. A pronounced increase in the total protein levels of ALK5 was detected at both 9 and 24 h in the pancreatitis group compared to sham control (Figure 2(h)). These data indicate that the acute pancreatitis mediated regulation of TGF-*β* responses at the receptor level predominantly involves induction of ALK5 instead of T*β*RII.

### 3.3. No Modulation of Smad2, 3, and 4 Levels in the Lungs

**Figure 3:**
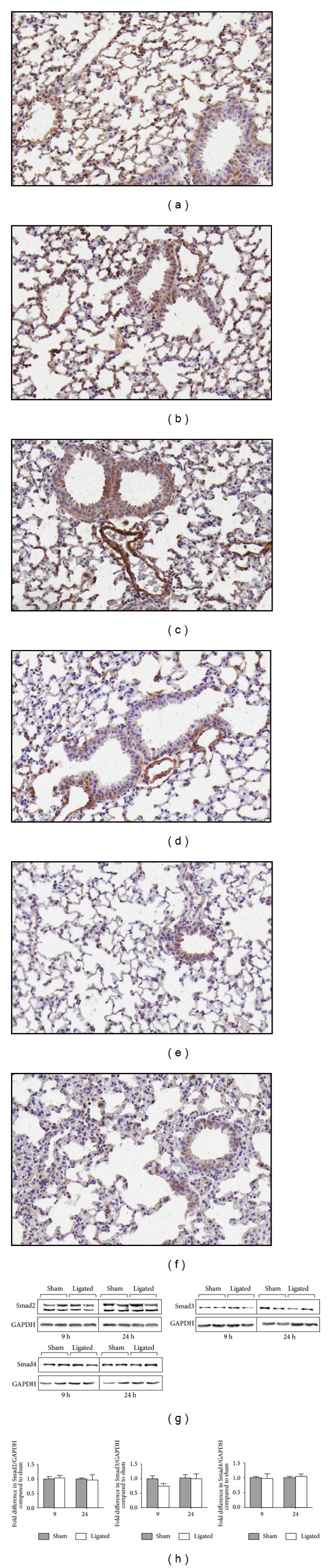
Distribution of Smad proteins in the lungs. Immunostaining of Smad2 (a and b), Smad3 (c and d), and Smad4 (e and f) levels in lung tissue at 9 h from sham controls (a, c, and e) and acute pancreatitis groups (b, d, and f). Results from one representative of eight samples per group are shown. Original magnification, ×20. Western blot analysis of Smad2, Smad3, and Smad4 protein levels in lung extracts of sham-operated and ligated animals at the indicated time points (g). Two of five similar analyses per group are shown. Graphs show densitometry measurements of fold difference in Smad2, Smad3, and Smad4 levels compared to sham control and represent mean ± SEM of five samples per group (h).

Next, modulation of the levels of Smad signaling effectors was investigated. Smad2 was detected at moderate levels in bronchial epithelium, infiltrating cells, and fibroblasts, while low levels were observed in alveoli and vascular endothelial cells in both sham controls and 9 h after acute pancreatitis induction (Figures 3(a) and 3(b)). High levels of Smad3 were observed in bronchial epithelium, vascular endothelium, infiltrating, and fibroblast-like cells, while low levels were detected in alveolar cells in both sham controls and with acute pancreatitis induction (Figures 3(c) and 3(d)). Similarly, lungs from both sham controls and acute pancreatitis-associated ALI showed moderate levels of Smad4 in bronchial epithelial, vascular endothelial and infiltrating cells (Figures 3(e) and 3(f)). A moderate Smad4 induction was noted in the infiltrating cells in the ligated animals compared to sham controls (Figure 3(f)). However, neither immunostainings nor Western blot (Figures 3(g) and 3(h)) analyses showed a significant modulation of the Smad2–4 protein levels in the lungs following 9 or 24 h acute pancreatitis induction compared to sham controls.

### 3.4. Transient Induction of Inhibitory Smad7 in the Lungs following Acute Pancreatitis

**Figure 4:**
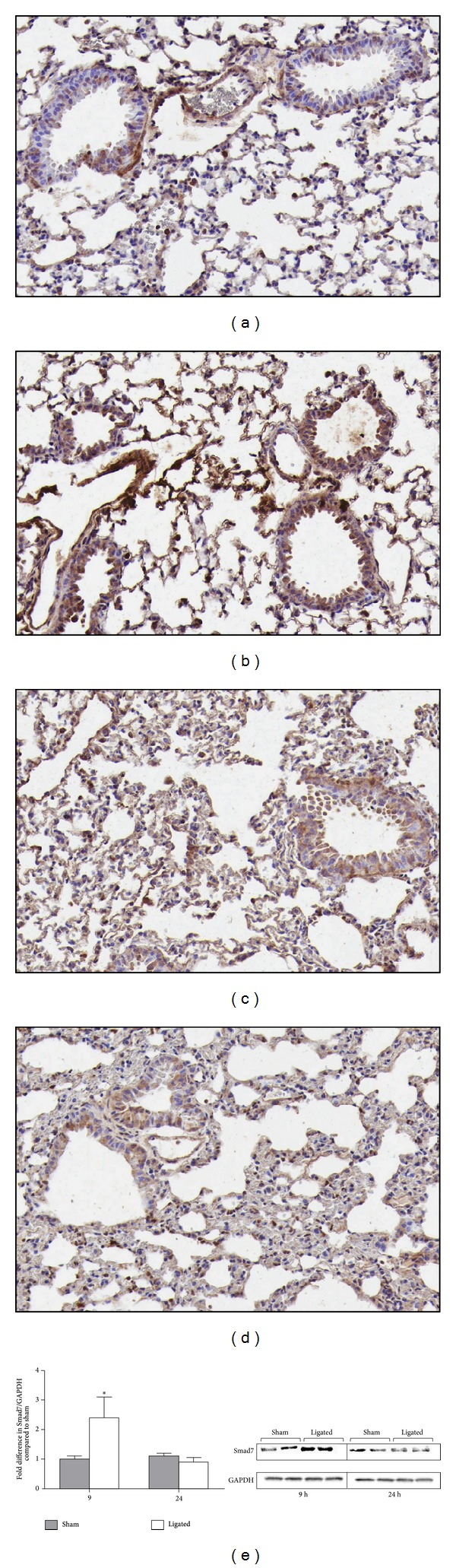
Transient induction of inhibitory Smad7 in the lungs. Representative images of Smad7 levels in lung tissue at 9 h (a and b) and 24 h (c and d) following sham operation (a and c) and acute pancreatitis induction (b and d). Results from one representative of eight samples per group are shown. Original magnification, ×20. Western blot analysis of Smad7 protein levels in lung extracts of sham-operated and ligated animals at two time points (e). Two of five similar analyses per group are shown. Graph displays densitometry measurement of fold difference in Smad7 levels compared to sham control and represents mean ± SEM of five samples per group (e). *P < 0.05.

To further study regulation of the TGF-*β* signaling pathway, levels of inhibitory Smad7 were evaluated. Bronchial epithelial cells as well as vascular endothelial, smooth muscle, and inflammatory cells showed considerably higher levels of Smad7 9 h after acute pancreatitis induction compared to sham controls (Figures 4(a) and 4(b)). However, the Smad7 induction appeared transient and with disease progression the levels of Smad7 were reduced. After 24 h the levels were not altered notably compared to control (Figures 4(c) and 4(d)). Analysis of the Smad7 protein levels in lung extracts confirmed the immunohistochemistry findings, with 2.41-fold increased levels of Smad7 (*P* < 0.05) in the ligated group at 9 h, but not at 24 h, compared to sham controls (Figure 4(e)).

### 3.5. Enhanced Nuclear Translocation of Phosphorylated Smad2 in the Ligated Animals

**Figure 5:**
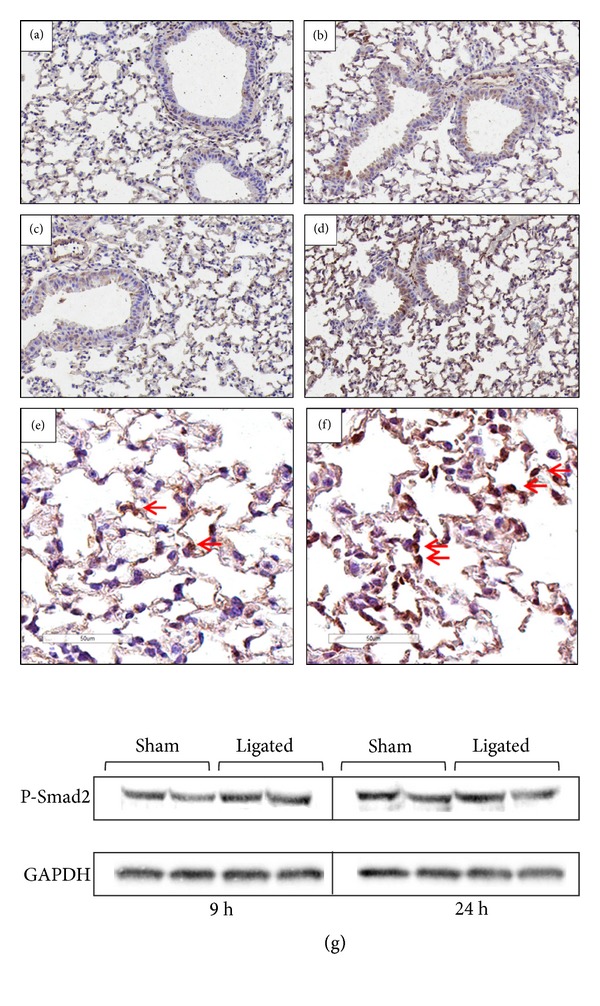
Redistribution and nuclear translocation of P-Smad2. Representative images of P-Smad2 in lung tissue at 9 h (a and b) and 24 h (c and d) following sham operation (a and c) and acute pancreatic induction (b and d). Original magnification, ×20. Arrows indicate nuclear translocation of P-Smad2 after 24 h in the ligated animals (f). More cytoplasmic (arrows) and less nuclear staining of P-Smad2 were observed in the sham-operated group (e). Original magnification, ×40. Results from one representative of eight samples per group are shown. Western blot analysis of total P-Smad2 protein levels in lung extracts of sham-operated and ligated animals (g). Two of five similar analyses per group are shown.

In order to study the level of active TGF-*β* signaling during acute pancreatitis-associated acute lung injury, the activation and subcellular distribution of phosphorylated Smad2 (P-Smad2) were evaluated in lung sections. Bronchial epithelial, vascular endothelial, infiltrating, and fibroblast-like cells in the sham-operated animals had a weak cytoplasmic expression of P-Smad2 at both 9 and 24 h, with scattered cells showing a nuclear localization (Figures 5(a) and 5(c)). In contrast, enhanced levels of cytoplasmic and importantly nuclear translocated P-Smad2 were detected in the lungs, especially in vascular endothelial cells, 24 h after acute pancreatitis induction compared to sham control (Figures 5(c)–5(f)). Despite differences at the cellular level, the total protein level of P-Smad2 was not altered between the sham-operated and ligated animals at any of the time points (Figure 5(g)).

A summary of the changes in expression of the TGF-*β* signaling molecules is presented in Table 1.

## 4. Discussion 

TGF-*β* has a well-established role in the fibrotic processes during chronic lung diseases. In the present study, we show that TGF-*β* signaling modulation starts as early as 9 h in the lungs of animals with acute pancreatitis-associated acute lung injury. These changes included an early increase in the levels of TGF-*β*1 and ALK5 (T*β*RI). A parallel increase in the signaling regulator and general inhibitor of the TGF-*β* signaling transduction, Smad7, was found at 9 h. Later in the course of the disease at 24 h the levels of TGF*β*1 and ALK5 remained high, while levels of the inhibitory Smad7 were reduced back to the level of the sham-operated animals. These changes were associated with an enhanced nuclear translocation of phosphorylated Smad2 indicating an active TGF-*β* signaling. This is the first report on the early modulation of TGF-*β* signaling pathway in the acute lung injury due to acute pancreatitis.

TGF-*β* signaling regulates various cellular processes, including cell proliferation, recognition, differentiation, apoptosis, and specification of developmental fate during embryogenesis as well as in mature tissues [5]. Previous studies have indicated that TGF-*β* not only participates in the late phase of acute lung injury, but also might be active early in acute lung injury and potentially could contribute to the development of pulmonary edema [22]. TGF-*β* mRNA level increased in murine intraparenchymal mononuclear cells and in alveolar macrophages within 1 h in a hemorrhage-induced acute lung injury model [23]. In addition to its classical role in fibrotic and extracellular matrix remodeling, TGF-*β* signaling has a pivotal role in angiogenesis and vascular defects. The *in vitro* effect of TGF-*β* on the integrity of vascular endothelium started within 1-2 h and was maximal 8-9 h after exposure to TGF-*β* [24]. TGF-*β*1-induced lung endothelial cell barrier dysfunction is mediated by activation of Rho and Rho-kinase, which induce endothelial cell permeability [25]. This effect appears to result from endothelial cell contraction through the activation of a myosin light chain kinase-dependent signaling cascade [24].

Our results showed an early increase (9 h) in the protein level of TGF-*β*1 in the lungs of animals with acute pancreatitis. This can be a contributing factor to the lung injury due to TGF-*β* effects on the vascular integrity. Endothelial damage facilitates inflammatory cell recruitment to the injured area. We have previously shown a significant enrichment of inflammatory cells (neutrophils and macrophages), recruited into the lung tissue of animals with acute pancreatitis at 9 h [21]. In addition to its effects on vascular permeability, TGF-*β*1 has been shown to enhance monocyte migration. TGF-*β*1-driven monocyte motility was mediated via ALK5-induced PI3 K and p38 signaling, independently of Smad2/3 [26]. The early rise in both TGF-*β*1 and ALK5 observed herein may thus contribute to stimulate the monocyte enrichment in the lungs at 9 h.

Disruption of the vascular integrity is not the only way that TGF-*β* signaling can play a role in the initiation and progression of acute lung injury. TGF-*β* activation plays a role in the development of acute lung injury through alteration of fibroproliferative responses, lung permeability, and inflammatory cell influx [18, 27–29]. TGF-*β* and TGF-*β*-inducible genes are able to modify lung permeability, epithelial ion transport, fibrinolysis, extracellular matrix, and surfactant homeostasis. The integration of the changes in these molecular pathways by TGF-*β* implicates it as a central mediator of acute lung injury [30]. In addition, TGF-*β* has potent proinflammatory and immune regulatory properties. Mice treated with anti-TGF-*β*antibodies showed decreased levels of pro-inflammatory cytokines compared to untreated one in a hemorrhage-induced acute lung injury model [23].

Different human diseases have been associated with dysregulation of TGF-*β* signaling, which shows the importance of a well-regulated signaling process. The TGF-*β*-Smad signaling pathway is regulated at different levels. Smad7 is part of an autoinhibitory feedback control, which interacts with ALK5 through various mechanisms. Smad7 activation is highly regulated and is induced by TGF-*β* stimulation [31]. Smad7 resides in the nucleus in unstimulated cells and translocates to the plasma membrane following receptor activation [32], where it binds the receptors and inhibits further signaling. Smad7 has a central role in pathological processes by its antifibrotic and anti-inflammatory properties [33]. The anti-inflammatory activities of increased Smad7 at early phase in our model can be interpreted as a protective mechanism in the lungs of the animals with acute pancreatitis.

In this study, we showed increased levels of both TGF-*β*1 and ALK5 in the lungs as early as 9 h after the induction of acute pancreatitis. However, increased TGF-*β* signaling via the Smad2 pathway was observed first after 24 h following acute pancreatitis induction. Increased nuclear translocation of phosphorylated Smad2 at 24 h can be due to the decreased Smad7 levels in the lungs. This time point was the peak of the inflammatory response associated with inflammatory cells recruitment to the lungs as well as pathological changes indicating the acute lung injury, in a previous report [21]. The later activation of the signaling pathway can be of interest in the acute lung injury, making the TGF-*β* pathway a potential therapeutic target. This is in accordance with previous report of attenuated lung injury by administration of a TGF-*β* inhibitor 24 h after the injury [18].

## 5. Conclusions

In summary, the present study shows an early rise in TGF-*β*1 and ALK5 which may activate the TGF-*β*/Smad2 signaling or alternative pathways to contribute to ALI. Acute lung injury is a critical syndrome due to a variety of precipitants, including acute pancreatitis. ALI remains a significant health burden with substantial morbidity and mortality. Improvements in outcome following ALI over the past decade are in part due to improved strategies of mechanical ventilation and advanced support of other failing organs and not a specific treatment. Clinical and experimental studies indicate a role of TGF-*β* in the early phases of ALI, where TGF-*β* contributes to the lung injury through epithelial and endothelial injuries. Our data showed an early rise in Smad7 which can block the TGF-*β*/Smad2 signaling. With disease progression, the Smad7 levels were reduced with a concomitant increase in nuclear translocation of phosphorylated Smad2, an indicator of active TGF-*β*/Smad2 signaling. An active TGF-*β* signaling can be a critical mediator for ALI.

## Figures and Tables

**Table 1:** Changes in expression patterns of TGF-*β* signaling molecules in the lungs during acute pancreatitis at two time points. The expression of TGF-*β* signaling molecules in the lungs of AP compared to sham-operated animals based on immunohistochemistry and Western blot analyses at 9 and 24 h. AP: acute pancreatitis.

	9 h	24 h
TGF-*β*1	↑ Expression in AP (*P* < 0.05)	↑↑ Expression in AP (*P* < 0.01)
TGF-*β*2 and 3	No difference between sham and AP	No difference between sham and AP
T*β*RII	No difference between sham and AP	No difference between sham and AP
ALK-5	↑ Expression in AP, ↑↑ positive cells in AP (*P* < 0.001)	↑↑ Expression in AP, ↑↑ positive cells in AP (*P* < 0.05)
Smad2, 3 and 4	No difference between sham and AP	No difference between sham and AP
Smad7	↑ Expression in AP (*P* < 0.05)	No difference between sham and AP
P-Smad2	No difference between sham and AP	↑ Nuclear translocation in AP

## Abbreviations

ALI: Acute lung injury
ALK: Activin like kinase
ARDS: Acute respiratory distress syndrome
TGF-*β*: Transforming growth factor-beta.
